# The optimal cut-off values of Klotho for predicting all-cause and cardiovascular mortality among chronic kidney disease: results from NHANES

**DOI:** 10.1038/s41598-024-52701-4

**Published:** 2024-02-26

**Authors:** Lili Liu, Junya Jia, Xi Cheng, Shan Gao, Tiekun Yan

**Affiliations:** Department of Nephrology, General Hospital of Tianjin Medical University, No. 154, Anshan Road, Heping District, Tianjin, 300052 China

**Keywords:** Biomarkers, Nephrology, Risk factors

## Abstract

To explore the optimal cut-off values of Klotho for predicting all-cause and cardiovascular mortality among chronic kidney disease (CKD) patients. Klotho was measured in 40–79-year-old individuals in the NHANES 2007–2016. A total of 2418 patients with stage 1–4 CKD were included. The optimal cut-off values of Klotho were utilized using receiver operator characteristic (ROC) curves and be verified on the effects of all-cause and cardiovascular mortality. Restricted cubic splines were used to examine the relationship between Klotho and all-cause and cardiovascular mortality with the optimal cutpoints as the reference. After a mean follow-up period of 87.9 months, 535 deaths occurred and 188 died of cardiovascular disease. Cubic splines showed that the risk of all-cause and cardiovascular mortality increased gradually for Klotho < 700 pg/ml. ROC curves revealed that the optimal cut-off values of Klotho for all-cause and cardiovascular mortality are 548.8 pg/ml and 660.9 pg/ml, respectively. Compared to patients with higher levels of Klotho, HRs (95% CIs) for all-cause and cardiovascular mortality were 1.52 (1.23, 1.87) and 1.58 (1.13, 2.22) among patients with lower levels of Klotho, respectively, in the multivariate model (*P* < .0001 and *P* = 0.008). Our findings revealed the optimal cut-off values of Klotho for all-cause and cardiovascular mortality in CKD.

## Introduction

Chronic kidney disease (CKD) is recognized as a public health problem, especially as its prevalence increases with the aging of the global population. CKD patients are at high risk of mortality. Cardiovascular disease (CVD) is the leading cause-of-death in patients with CKD. Even mild renal impairments increase the risk of CVD and death, and the risk increases gradually as renal function deteriorates^1^.

Alpha Klotho was first identified as an anti-aging protein in 1997. A deficiency in the Klotho gene can cause aging in mice, including atherosclerosis and osteoporosis^2^. Klotho is highly expressed in renal tubular cells as a transmembrane protein, and its expression declines in the early stages of CKD^3^. Soluble Klotho is derived from the cleavage of the membranous Klotho protein, thus may reflect the senescence of the kidney. Soluble Klotho is a pleiotropic protein that acts as a paracrine and endocrine hormonal factor in multiple organs. It binds to fibroblast growth factor 23 (FGF23) and functions as the modulation of mineral homeostasis^4^. It is a critical protein that protects the kidney, and it has been proven to delay CKD progression in animal experiments^5,6^ and population study^7^. Postulated mechanisms include the suppression of oxidative stress and inflammation, the activation of autophagy, and the mitigation of mitochondrial damage^8^. Given the importance of Klotho protein, it is necessary to understand its cut-off value. However, limited evidence on the cut-off values of Klotho for clinical outcomes has been reported in CKD patients.

There is emerging observational evidence that lower serum Klotho levels are associated with increased risk of death in individuals in the general population^9^, hypertension^10^, and hemodialysis^11–13^. Previous studies reported that lower Klotho levels are associated with increased risk of adverse outcomes only based on quantiles of Klotho without reporting a threshold. Furthermore, the effect of serum Klotho on adverse outcomes in CKD patients still remains controversial: a cohort study conducted on 312 patients with stage 2–4 CKD revealed that plasma levels of soluble Klotho were not associated with kidney function and did not predict death or the initiation of renal replacement therapy after 26 months of follow-up^14^; while another observational study including 243 stage 1–5 CKD patients showed that lower circulating Klotho levels were associated with the doubling of serum creatinine concentrations, end-stage kidney disease (ESKD), or death after 29.7 months of follow-up^15^. The possible explanations underlying the conflicting results may be the heterogeneity of study design including relatively small number of patients, diverse outcomes as well as the different reference values due to the different grouping methods, such as median or tertile. There is reason to believe that those simple classification methods do not capture the optimal cut-off value. The lack of optimal cut-off value also brings confusion to the clinical practice. Studies with a larger sample sizes and longer follow-up periods regarding Klotho with a threshold on adverse outcomes including all-cause and cardiovascular mortality in pre-dialysis CKD patients are limited and warranted^15,16^. The National Health and Nutrition Examination Survey (NHANES) is a national cross-sectional study in America (with a nationally representative population) and linked to the National Death Index. Therefore, we aimed to explore the cut-off values of serum soluble Klotho on adverse outcomes (all-cause and cardiovascular mortality) in patients with pre-dialysis CKD by using the NHANES database.

## Materials and methods

### Study population

**Figure 1:**
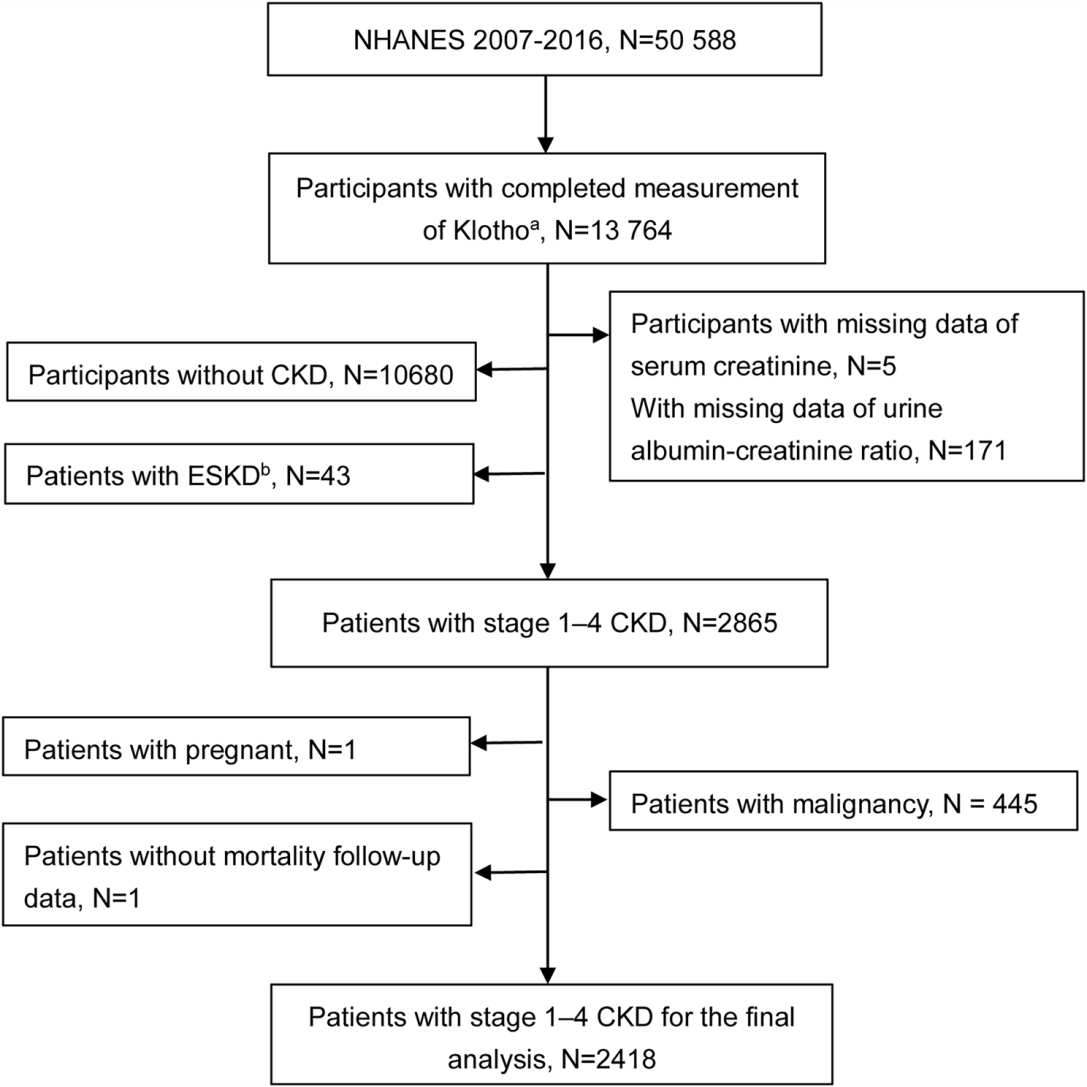
Flowchart of the participant inclusion process. *Note*: ^a^Klotho was measured in participants aged 40–79 years. ^b^ESKD: end-stage kidney disease, which is defined as eGFR < 15 ml/min/1.73m^2^ and/or dialysis.

NHANES is a nationally representative sample of the civilian, non-institutionalized American population obtained via stratified, complex multistage probability sampling. It monitors the health and nutritional statuses of people across the United States. In our analyses, participants were derived from five consecutive waves of NHANES (2007–2008, 2009–2010, 2011–2012, 2013–2014, 2015–2016) (N = 50 588) since Klotho was available during these survey waves. We initially restricted our analyses to subsamples with complete serum Klotho measurements because Klotho was only measured in individuals aged 40–79 years. A total of 2418 individuals with stage 1–4 CKD were included in the current analysis. The detailed flow chart of the participant recruitment process is shown in Fig. 1. The collection of NHANES data was approved by the National Center for Health Statistics ethics review board. All participants gave their written informed consent.

### Definition of CKD

Serum or urinary creatinine levels were determined via the Jaffe rate method. Urinary albumin was tested using the solid-phase fluorescent immunoassay method. We calculated the estimated glomerular filtration rate (eGFR) using the CKD Epidemiology Collaboration (CKD-EPI) equation based on serum creatinine^17^. We also calculated the urine albumin-creatinine ratio (ACR). CKD was defined as eGFR < 60 ml/min/1.73 m^2^ or ACR ≥ 30 mg/g. CKD stages were classified based on previously published guidelines as follows: stage 1 (eGFR ≥ 90 ml/min/1.73 m^2^ and ACR ≥ 30 mg/g), stage 2 (eGFR 60–89 ml/min/1.73 m^2^ and ACR ≥ 30 mg/g), stage 3 (eGFR 30–59 ml/min/1.73 m^2^), and stage 4 (eGFR 15–29 ml/min/1.73 m^2^)^18^.

### Measurements of serum Klotho

During 2019, Klotho was measured in 40–79-year-old participants in NHANES 2007–2016 who gave their consent for their samples to be used in future research using a commercially available ELISA kit (IBL International, Japan). All samples were stored at – 80 °C until predefined batches of samples were provided daily to the technicians for analyses. Samples were received on dry ice and analyzed in duplicate, and the average of the two values was used. If the difference between the two values exceeded 10%, analyses were repeated.

### Outcomes

The main outcomes were all-cause and cardiovascular mortality. All participants in each of the survey cycles were linked to the National Death Index until December 31, 2019, which captures the vital status and cause-of-death. The cause-of-death was identified according to the International Classification of Diseases, 10th Edition (ICD-10), and cardiovascular mortality codes I00–I09, I11, I13, I20–I51, and I60-I69.

### Assessment of covariates

Patients’ demographic data included age, sex, and race/ethnicity. Self-reported living habits included cigarette smoking and physical activity. Those who had smoked fewer than 100 cigarettes in their lifetime were classified as never-smokers. Those who had smoked at least 100 cigarettes in their lifetime but were not currently smoking or currently smoking some days/every day were classified as former smokers and current smokers, respectively. According to physical activity, patients were divided into two categories based on whether or not they met national guidelines of ≥ 150 min/week of moderate activity or ≥ 75 min/week of vigorous activity^19^. Anthropometric indicators included the body mass index (BMI) and blood pressure. BMI was calculated as weight in kilograms divided by the square of the height in meters. Blood pressure was measured three times after the patient had rested quietly in the sitting position for 5 min, and the mean value was used for analyses. Comorbidities included hypertension, diabetes, and CVD. Hypertension was defined as a self-reported history of hypertension and/or systolic blood pressure (SBP) ≥ 140 mmHg and/or diastolic blood pressure (DBP) ≥ 90 mmHg and/or the use of hypotensive agents. Diabetes was defined as a self-reported history of diabetes and/or fasting blood glucose ≥ 7.0 mmol/L and/or hemoglobin A1c (HbA1c) ≥ 6.5% and/or the use of hypoglycemic agents. CVD was defined as a history of coronary artery disease, angina, heart attack, heart failure, or stroke. Laboratory data, including high-density lipoprotein cholesterol (HDL-C), triglycerides, albumin-corrected calcium, phosphate, and 25-hydroxyvitamin D (25(OH)D), were measured as previously described^20^.

### Statistical analysis

The baseline characteristics of participants were described and compared across quartiles of Klotho. Continuous variables were presented as the mean ± SD or the median (interquartile range, IQR). Categorical variables were expressed as frequencies (percentages).

To visualize the distribution of Klotho, we plotted its distribution in the entire patient population and examined its trends across CKD stages. Cox proportional hazards models and competing risk models were used to assess the relationship between quartiles of Klotho and all-cause and cardiovascular mortality using the highest quartile of Klotho as the reference, and hazard ratios (HRs) and 95% confidence intervals (CIs) were calculated. Because the number of missing data was very small, they were either imputed as mean/median values or recoded before Cox regression. The details of missing data are displayed in the footnote of Table 1. We initially constructed a crude model (unadjusted model) and subsequently constructed multivariable models. Model 1 was adjusted for age, sex, and race/ethnicity. Model 2 was additionally adjusted for smoking (never, former, and current), physical activity (meet the suggested guideline or not), obesity (defined as BMI > 30 kg/m^2^), HbA1c (> 6.5% or not), diabetes, hypertension, CVD, hypotensive agents, hypoglycemic agents, HDL-C, and triglycerides. Model 3 was additionally adjusted for calcium, phosphate and 25(OH)D, and the CKD stage. Receiver operator characteristic (ROC) curves were utilized to choose the optimal cut-off values of Klotho for all-cause and cardiovascular mortality. ROC curve is a common method for calculating cutoff points, and it can be derived after adjusting other covariates^21^ by plotting the sensitivity on the y axis against 1-specificity on the x axis. Two methods commonly used to establish the optimal cut-off point include the point on the ROC curve closest to (0, 1) and the measurement of the biggest Youden index, i.e., sensitivity + specificity - 1^22^. It have a distinct advantage that the performance of the model can be examined using the area under the ROC curve (AUC). The covariates included in the ROC curve were the confounders in model 3 as described above. The dose–response relationship between Klotho and all-cause and cardiovascular mortality was evaluated by using restricted cubic splines with knots of the 20th, 40th, 60th, and 80th percentiles and the optimal cutpoints of Klotho as the reference. The same potential confounding factors in the fully adjusted Cox model were included in restricted cubic spline models. To verify the effects of the chosen cut-off values of Klotho, we conducted further analysis. Cumulative Kaplan–Meier curves were plotted for all-cause and cardiovascular mortality, and the log-rank test was used to evaluate the statistical significance. Cumulative hazard plots indicated no appreciable violations of the proportional hazards. The association between the optimal cutpoints of Klotho and all-cause and cardiovascular mortality were evaluated using Cox proportional hazards models and competing risk models, respectively, in stepwise multivariable models. Sensitivity analyses were repeated after excluding patients who died during the first two years of follow-up. Thereafter, we evaluated if the association between Klotho and mortality was modified by age, sex, physical activity, smoking, obesity, diabetes, hypertension, CVD, and CKD stages by including interaction terms for each in the final multivariable models. Analyses were conducted by using SAS System version 9.4 (SAS Institute; Cary, NC), Graphpad Prism 6.0 software, and Stata/MP version 14.0 for Windows (Stata Corp., College Station, Texas, USA), A two-sided P-value of < 0.05 was considered statistically significant.

**Table 1:** Characteristics of patients with stage 1–4 CKD according to quartiles of serum Klotho levels.

	Total	Quartiles of serum Klotho levels, pg/ml			
		Quartile 1	Quartile 2	Quartile 3	Quartile 4
		< 622	622–761	761–968	> 968
Number	2418	605	605	605	603
Age (years)	62.4 ± 10.7	63.9 ± 10.2	63.3 ± 10.5	62.5 ± 10.3	59.9 ± 11.1
Male (n (%))	1169 (48.4)	307 (50.7)	304 (50.3)	293 (48.4)	265 (43.9)
Race (n (%))					
Non-Hispanic white	886 (36.6)	235 (38.8)	251 (41.5)	225 (37.2)	175 (29.0)
Non-Hispanic black	717 (29.7)	194 (32.1)	147 (24.3)	173 (28.6)	203 (33.7)
Hispanic	246 (10.2)	50 (8.3)	54 (8.9)	70 (11.6)	72 (11.9)
Others	569 (23.5)	126 (20.8)	153 (25.3)	137 (22.6)	153 (25.4)
Smoking (n (%))					
Never	1160 (48.0)	253 (42.0)	263 (43.5)	306 (50.6)	338 (56.0)
Former	760 (31.5)	218 (36.2)	207 (34.2)	185 (30.6)	150 (24.9)
Current	495 (20.5)	131 (21.8)	135 (22.3)	114 (18.8)	115 (19.1)
Drinking (n (%))	1493 (66.0)	410 (72.1)	376 (65.4)	375 (66.5)	332 (59.9)
Physical activity meets the guidelines (n (%))	1250 (51.7)	293 (48.4)	312 (51.6)	327 (54.1)	318 (52.9)
SBP (mmHg)	134.7 ± 21.7	134.1 ± 20.3	135.4 ± 22.3	135.5 ± 22.7	133.8 ± 21.6
DBP (mmHg)	71.0 ± 15.1	68.6 ± 15.6	71.2 ± 15.8	71.9 ± 14.4	72.4 ± 14.5
BMI (kg/m^2^)	31.1 ± 7.4	31.3 ± 7.1	31.0 ± 7.0	31.1 ± 7.9	31 ± 7.6
Serum Klotho (pg/ml)	826.8 ± 307.3	514.6 ± 78.4	691.5 ± 40.4	856.5 ± 59.5	1246.1 ± 272.8
Calcium (mmol/l)	2.4 ± 0.1	2.4 ± 0.1	2.4 ± 0.1	2.4 ± 0.1	2.4 ± 0.1
Phosphorus (mmol/l)	1.2 ± 0.2	1.2 ± 0.2	1.2 ± 0.2	1.2 ± 0.2	1.2 ± 0.2
25(OH) vitamin D (nmol/l)	65.3 ± 29.7	68.1 ± 32.3	67.8 ± 29.5	64.4 ± 30.4	60.7 ± 25.7
eGFR (ml/min/1.73m^2^)	69.3 ± 24.3	63.1 ± 22.8	66.9 ± 24.5	70.5 ± 23.2	76.6 ± 24.8
ACR (mg/g)	42.8 (11.6,103.8)	39.5 (8.3,98.6)	40.7 (11.2,113.2)	41.7 (12.6,97.2)	47.4 (26.2,112.8)
CKD stages					
Stage 1	610 (25.2)	99 (16.4)	142 (23.5)	155 (25.6)	214 (35.5)
Stage 2	636 (26.3)	144 (23.8)	140 (23.1)	178 (29.4)	174 (28.9)
Stage 3	1097 (45.4)	335 (55.4)	296 (48.9)	259 (42.8)	207 (34.3)
Stage 4	75 (3.1)	27 (4.4)	27 (4.5)	13 (2.2)	8 (1.3)
Total cholesterol (mmol/l)	5.0 ± 1.2	5.0 ± 1.2	5.0 ± 1.2	5.0 ± 1.2	5.1 ± 1.2
HDL-C (mmol/l)	1.3 ± 0.4	1.4 ± 0.5	1.3 ± 0.4	1.3 ± 0.4	1.3 ± 0.5
Triglyceride (mmol/l)	1.6 (1.1, 2.5)	1.7 (1.2,2.5)	1.7 (1.2,2.6)	1.5 (1,2.5)	1.7 (1,2.6)
Fasting blood glucose (mmol/l)	7.3 ± 3.4	6.7 ± 2.2	7.0 ± 2.8	7.0 ± 3.0	8.6 ± 4.8
HbA1c (%)	6.5 ± 1.7	6.3 ± 1.2	6.4 ± 1.4	6.5 ± 1.6	7.0 ± 2.3
Diabetes (n (%))	1044 (43.2)	261 (43.1)	236 (39.0)	249 (41.2)	298 (49.4)
Hypertension (n (%))	1818 (75.2)	473 (78.2)	466 (77.0)	458 (75.7)	421 (69.8)
Cardiovascular disease (n (%))	576 (23.8)	166 (27.4)	155 (25.6)	139 (23.0)	116 (19.2)
Hypotensive drugs (n (%))	1507 (62.3)	408 (27.1)	373 (24.8)	382 (25.4)	344 (22.8)
Hypoglycemic drugs (n (%))	781 (32.3)	218 (36.0)	173 (28.6)	184 (30.4)	206 (34.2)

*Note: CKD* chronic kidney disease, *SBP* systolic blood pressure, *DBP* diastolic blood pressure, *BMI* body mass index, *eGFR* estimated glomerular filtration rate, *CVD* cardiovascular disease, *ACR* urine albumin-creatinine ratio, *HDL-C* high-density lipoprotein cholesterol, *HbA1c* hemoglobin A1c.

Missing data: Smoking: 3; Physical activity: 2; SBP: 69; DBP: 69; BMI: 50; Calcium: 2; Phosphorus: 1; 25(OH) vitamin D: 25; Triglycerides: 2; HbA1c: 4.

## Results

### Baseline characteristics of patients

A total of 2418 patients with stage 1–4 CKD were included in the current analysis. The mean age of our participants was 62.4 ± 10.7 years, 1169 (48.4%) participants were male, and 36.6% of them were non-Hispanic white. The mean Klotho was 826.8 ± 307.3 pg/ml. The mean eGFR was 69.3 ± 24.3 mL/min/1.73 m^2^, and the median ACR was 42.8 mg/g (IQR: 11.6, 103.8), and 25.2%, 26.3%, 45.4% and 3.1% of participants were at CKD stage 1–4, respectively. As shown in Table 1, patients with lower Klotho levels are more likely to be older patients, to be smokers, to have hypertension and CVD, to be less likely to meet suggested physical activity guidelines, and to have higher 25(OH)D levels and lower DBP, eGFR, fasting glucose or HbA1c levels. During follow-up, 535 deaths were recorded. A total of 188 patients died of CVD (Table 2).

**Table 2:** Hazard ratios (95% CIs) for the association between serum Klotho levels and all-cause and cardiovascular mortality.

	Quartile 1	Quartile 2	Quartile 3	Quartile 4
All-cause mortality: 535 (22.1%)				
N (%)	171 (28.3)	138 (22.8)	121 (20.0)	105 (17.4)
Unadjusted	1.76 (1.38, 2.24)	1.36 (1.06, 1.76)	1.20 (0.92, 1.56)	1.0
Model 1	1.39 (1.09, 1.78)	1.09 (0.85, 1.41)	1.03 (0.79, 1.34)	1.0
Model 2	1.35 (1.05, 1.73)	1.03 (0.80, 1.34)	1.03 (0.79, 1.35)	1.0
Model 3	1.34 (1.04, 1.72)	1.03 (0.79, 1.33)	1.01 (0.77, 1.31)	1.0
Cardiovascular mortality: 188 (7.8%)				
N (%)	67 (11.1)	41 (6.8)	45 (7.4)	35 (5.8)
Unadjusted	1.97 (1.31, 2.96)	1.18 (0.75, 1.85)	1.31 (0.85, 2.04)	1.0
Model 1	1.61 (1.06, 2.44)	1.00 (0.63 1.58)	1.17 (0.75, 1.82)	1.0
Model 2	1.56 (1.02, 2.38)	0.91 (0.57, 1.47)	1.15 (0.73, 1.81)	1.0
Model 3	1.52 (0.99, 2.33)	0.89 (0.55, 1.44)	1.06 (0.67, 1.68)	1.0

*Note:* Quartile 1: < 622 pg/ml; Quartile 2: 622–761 pg/ml; Quartile 3: 761–968 pg/ml; Quartile 4: > 968 pg/ml; Model 1 was adjusted for age, sex, race/ethnicity. Model 2 was additionally adjusted for smoking (never: former and current), physical activity (meet the suggested guideline or not), obesity (defined as BMI > 30 kg/m^2^ or not), HbA1c (> 6.5% or not), diabetes, hypertension, CVD, hypotensive agents, hypoglycemic agents, HDL-C and triglyceride. Model 3 was additionally adjusted for calcium, phosphate and 25(OH)D and CKD stages.

### Distribution of Klotho and trends across CKD stages

**Figure 2:**
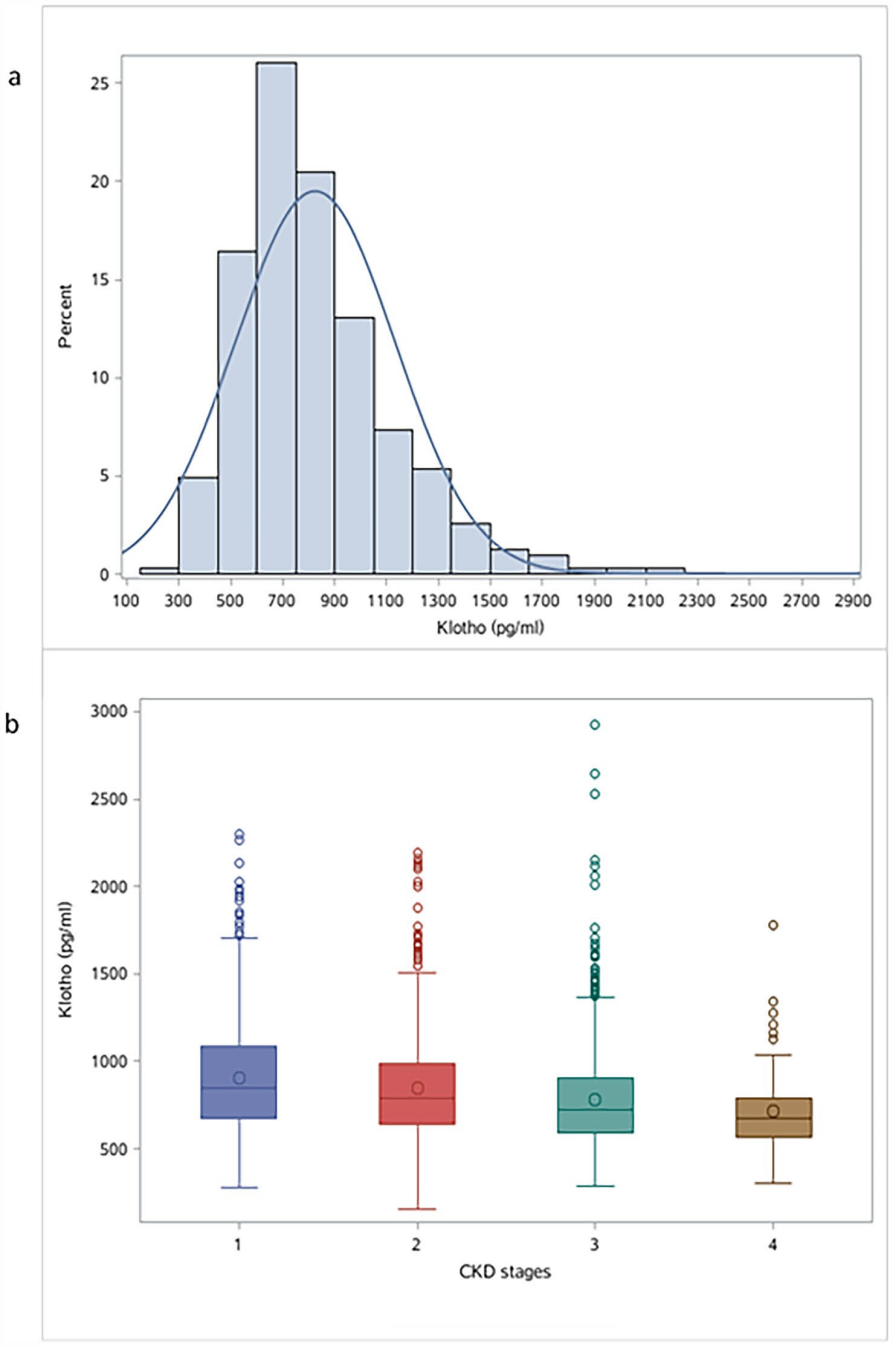
Distribution of Klotho in all patients and trends stratified by CKD stages. *Note: CKD* chronic kidney disease.

The Klotho concentration ranged from 152.5 pg/ml to 2927.3 pg/ml with a roughly normal distribution. Klotho concentrations gradually decreased with the CKD stages (Fig. 2).

### Klotho and outcomes

#### *All-cause mortality*

The mean follow-up for the cohort was 87.9 months. During follow-up, 535 deaths were recorded. Among those cases, 171 (28.3%), 138 (22.8%), 121 (20.0%), and 105 (17.4%) deaths were identified in Klotho quartiles 1, 2, 3, and 4, respectively (Table 2). When Klotho was examined in quartiles, the association with all-cause mortality remained significant and did not change significantly in all multivariable models, with the lowest quartile indicating a 34% higher risk of all-cause mortality compared with the highest quartile after adjustment for demographics, living habits, CVD risk factors, biomarkers of mineral metabolism, and CKD stages (Table 2).

#### *Cardiovascular mortality*

A total of 188 patients died of CVD. Among those cases, 67 (11.1%), 41 (6.8%), 45 (7.4%), and 35 (5.8%) were recorded across Klotho quartiles 1, 2, 3, and 4, respectively (Table 2). In categorical analyses, the lowest quartile of Klotho was associated with a higher risk of cardiovascular mortality (HR 1.56 (95% CI: 1.02, 2.38)) before adjusting for calcium, phosphate, 25(OH)D and CKD stages, compared with the highest quartile. However, this tendency did not reach statistical significance in stepwise multivariable models (Table 2).

### The optimal cut-off values of Klotho

**Figure 3:**
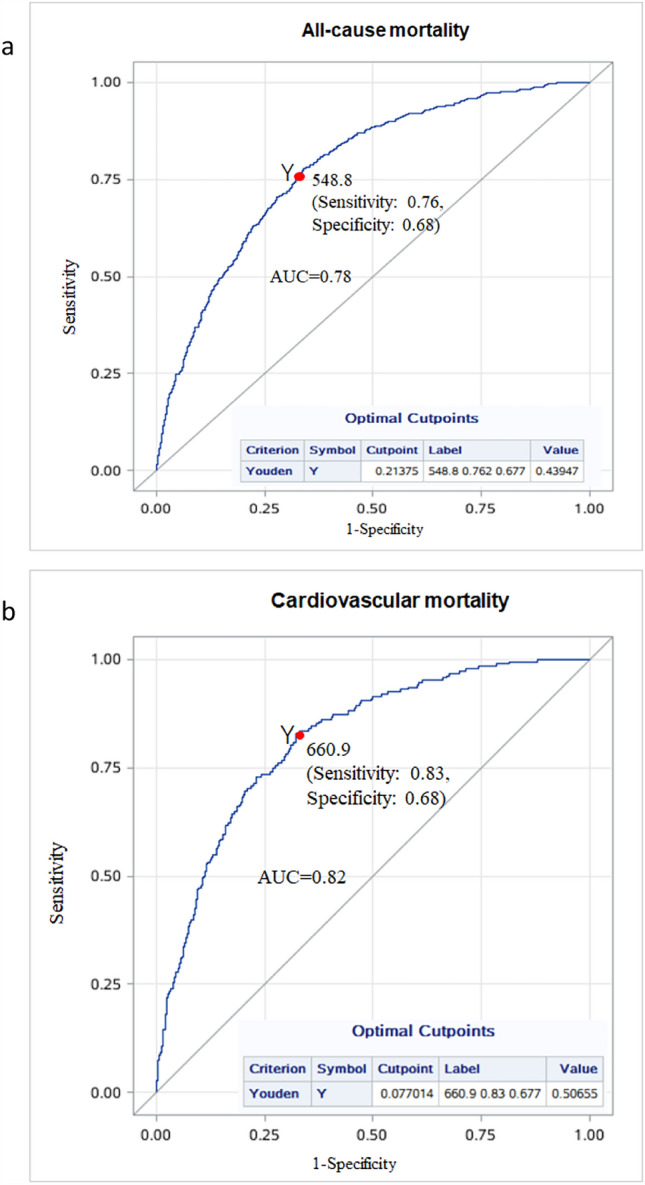
The optimal cutoff points of Klotho for all-cause and cardiovascular mortality using ROC curves. *Note*: “Label” represents the optimal cutpoint of Klotho, sensitivity and specificity. “Value” represents the youden index (i.e., sensitivity + specificity-1).

The ROC curves were utilized to choose the optimal cut-off values of Klotho for all-cause and cardiovascular mortality after including the above covariates in Model 3. Our results indicated that the optimal cut-off values of Klotho for predicting all-cause and cardiovascular mortality were 548.8 pg/ml and 660.9 pg/ml, respectively, and the sensitivity were 76% and 83%, respectively; the specificity were both 68%. The AUC were 0.78 and 0.82, respectively (Fig. 3).

### Cubic splines

**Figure 4:**
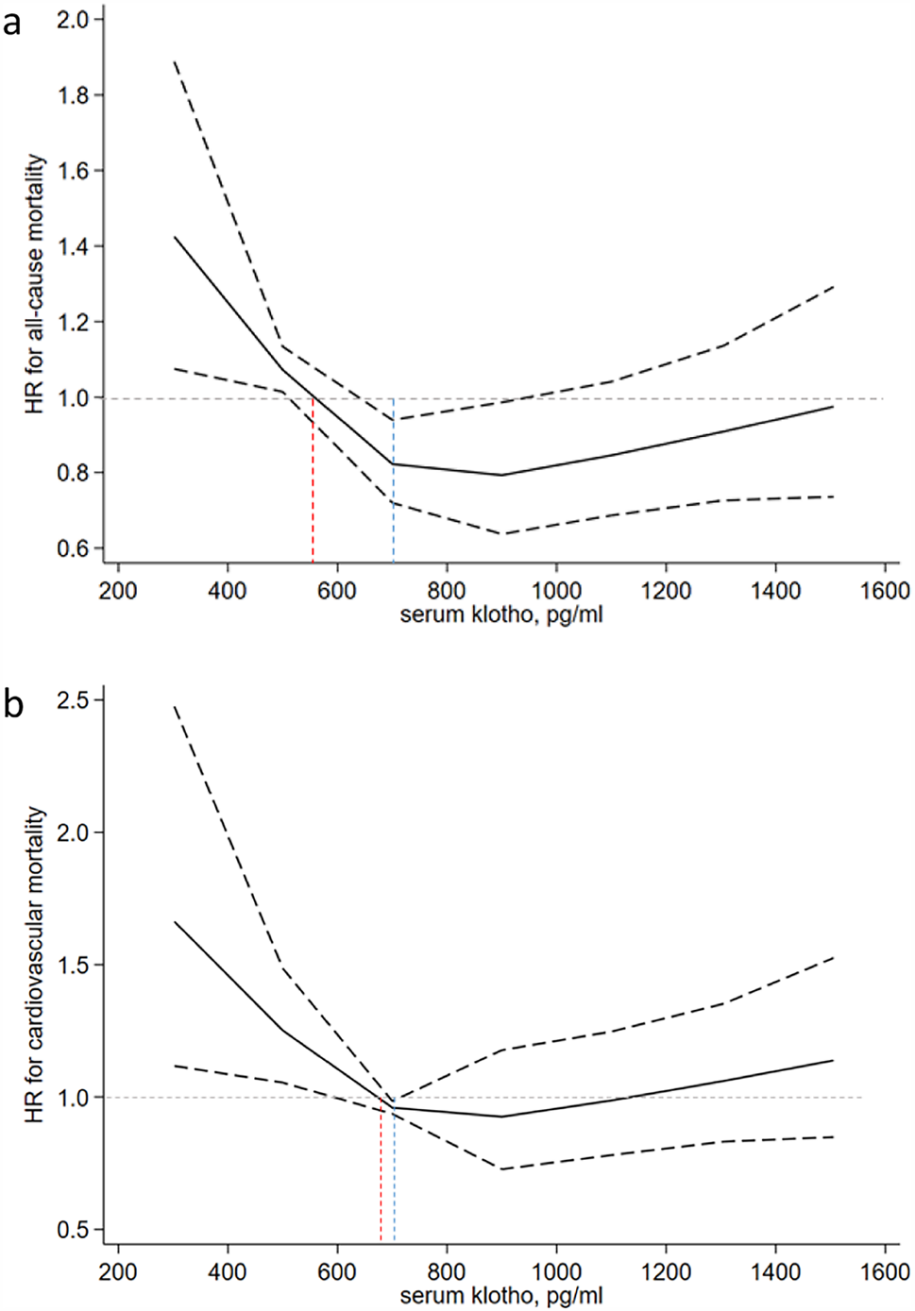
Dose–response relationship between Klotho and all-cause and cardiovascular mortality. *Note*: The reference values for each hazard ratio were Klotho = 548.8 pg/ml and 660.9 pg/ml for all-cause and cardiovascular mortality, respectively (red dotted line). The solid line represents the estimated hazard ratio and the dotted line represents the 95% confidence interval. The range of Klotho in the curve was restricted because the majority of patients (96.3%) had Klotho concentrations that ranged between 300 pg/ml and 1500 pg/ml.

To visualize the dose–response relationship of Klotho and all-cause and cardiovascular mortality, the restricted cubic splines were plotted with the optimal cutoff points as the reference. In spline analyses, we observed a consistent pattern for all-cause and cardiovascular mortality. The range of Klotho in the curve was restricted because the majority of patients (96.3%) had Klotho concentrations between 300 pg/ml and 1500 pg/ml. As shown in Fig. 4, a non-linear association was observed between Klotho and all-cause and cardiovascular mortality. The reference values for each hazard ratio were Klotho = 548.8 pg/ml and 660.9 pg/ml for all-cause and cardiovascular mortality, respectively (the red dotted line). When Klotho concentrations were below 700 pg/ml (the blue dotted line), the risk of all-cause and cardiovascular mortality gradually increased as the Klotho concentration decreased.

### The optimal cutpoints of Klotho and outcomes

To verify the effects of the chosen cut-off values, we stratified patients into two groups (Klotho < 548.8 pg/ml, N = 366 (15.1%) vs. ≥ 548.8 pg/ml, N = 2052 (84.9%) and Klotho < 660.9 pg/ml, N = 408 (16.9%) vs. ≥ 660.9 pg/ml, N = 2010 (83.1%) for further analysis.

#### *All-cause mortality*

**Figure 5:**
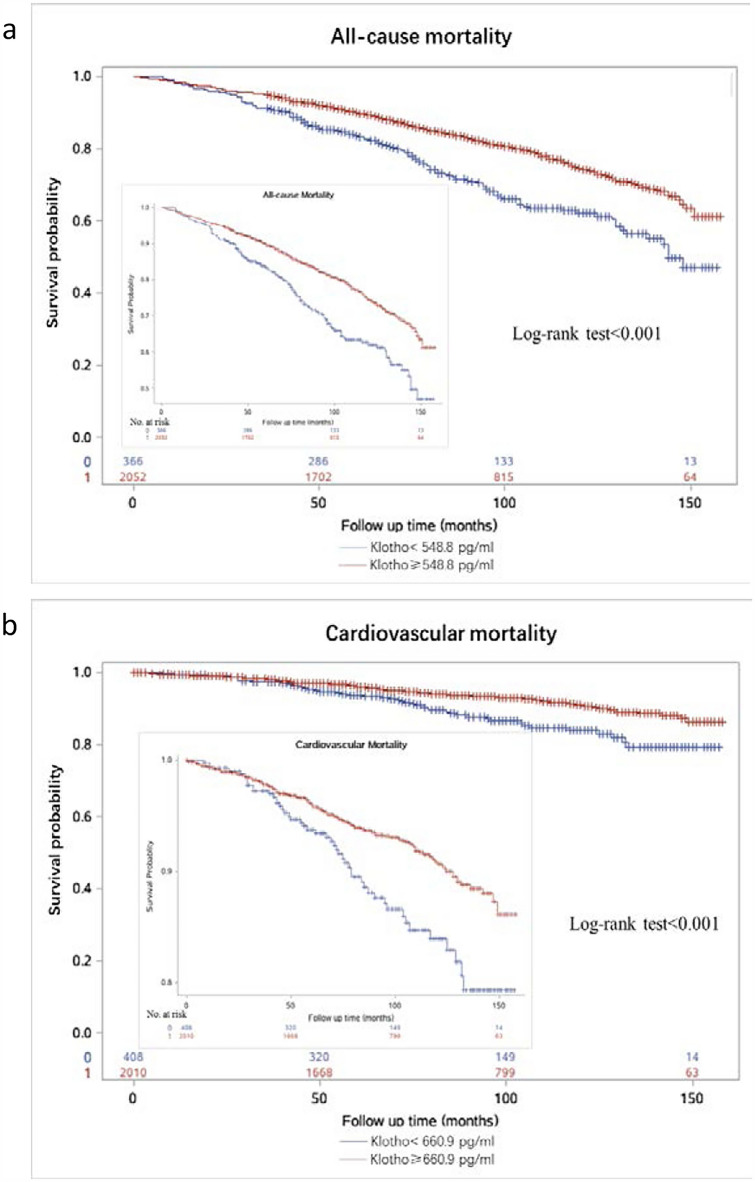
The Kaplan–Meier curves for the optimal cutoff points of Klotho and all-cause and cardiovascular mortality.

Among 535 deaths, 120 (32.8%) and 415 (20.2%) deaths were identified in patients with Klotho < 548.8 pg/ml and those ≥ 548.8 pg/ml, respectively. Kaplan–Meier survival curves were shown in Fig. 5a. Participants with serum Klotho in higher levels had significantly higher overall survival compared to those with lower Klotho levels (log-rank test, P < 0.001). Similar findings were observed when Klotho was examined in Cox regression, patients with Klotho < 548.8 pg/ml indicating a higher risk of all-cause mortality compared with those ≥ 548.8 pg/ml. This association remained significant in all multivariable models (HR and 95% CI 1.52 (1.23, 1.87), P < 0.0001) (Table 3).

**Table 3:** Hazard ratios (95% CIs) for the associations between Klotho and all-cause and cardiovascular mortality.

	All-cause mortality	Cardiovascular mortality
Unadjusted		
HR	1.69 (1.38, 2.07)	1.76 (1.27, 2.44)
* P*	< 0.0001	< 0.001
Model 1		
HR	1.53 (1.25, 1.88)	1.59 (1.14, 2.21)
*P*	< 0.0001	0.006
Model 2		
HR	1.51 (1.23, 1.86)	1.60 (1.15, 2.22)
*P*	< 0.0001	0.005
Model 3		
HR	1.52 (1.23, 1.87)	1.58 (1.13, 2.22)
*P*	< 0.0001	0.008
Senstivity analysis		
HR	1.62 (1.29, 2.03)	1.78 (1.24, 2.55)
*P*	< 0.0001	0.002

*Note*: The cut-off values of Klotho are 548.8 pg/ml for all-cause mortality and 660.9 pg/ml for cardiovascular mortality, respectively. Model 1 was adjusted for age, sex, race/ethnicity. Model 2 was additionally adjusted for smoking (never: former and current), physical activity (meet the suggested guideline or not), obesity (defined as BMI > 30 kg/m^2^ or not), HbA1c (> 6.5% or not), diabetes, hypertension, CVD, hypotensive agents, hypoglycemic agents, HDL-C and triglyceride. Model 3 was additionally adjusted for calcium, phosphate and 25(OH)D and CKD stages. Senstivity analysis included confounders in model 3.

#### *Cardiovascular mortality*

Among those cases, 48 (11.8%) and 140 (7.0%) cases were recorded in Klotho < 660.9 pg/ml and ≥ 660.9 pg/ml, respectively. Kaplan–Meier survival curves are shown in Fig. 5b. Participants with serum Klotho in lower level had significantly higher risk of cardivascualr mortality compared to those with higher Klotho levels (log-rank test, P < 0.001). In categorical analyses, the risk of cardiovascular mortality is higher in patients with Klotho < 660.9 pg/ml compared with those ≥ 660.9 pg/ml (HR and 95% CI: 1.58 (1.13, 2.22), *P* = 0.008) in the fully adjusted model (Table 3).

#### *Sensitivity analysis*

Eighty-eight participants were excluded from the analysis because they died during the first two years of follow-up, leaving 2330 participants available for the analysis. The association did not change significantly in the sensitivity analysis (Table 3).

### Interaction and subgroup analysis

**Figure 6:**
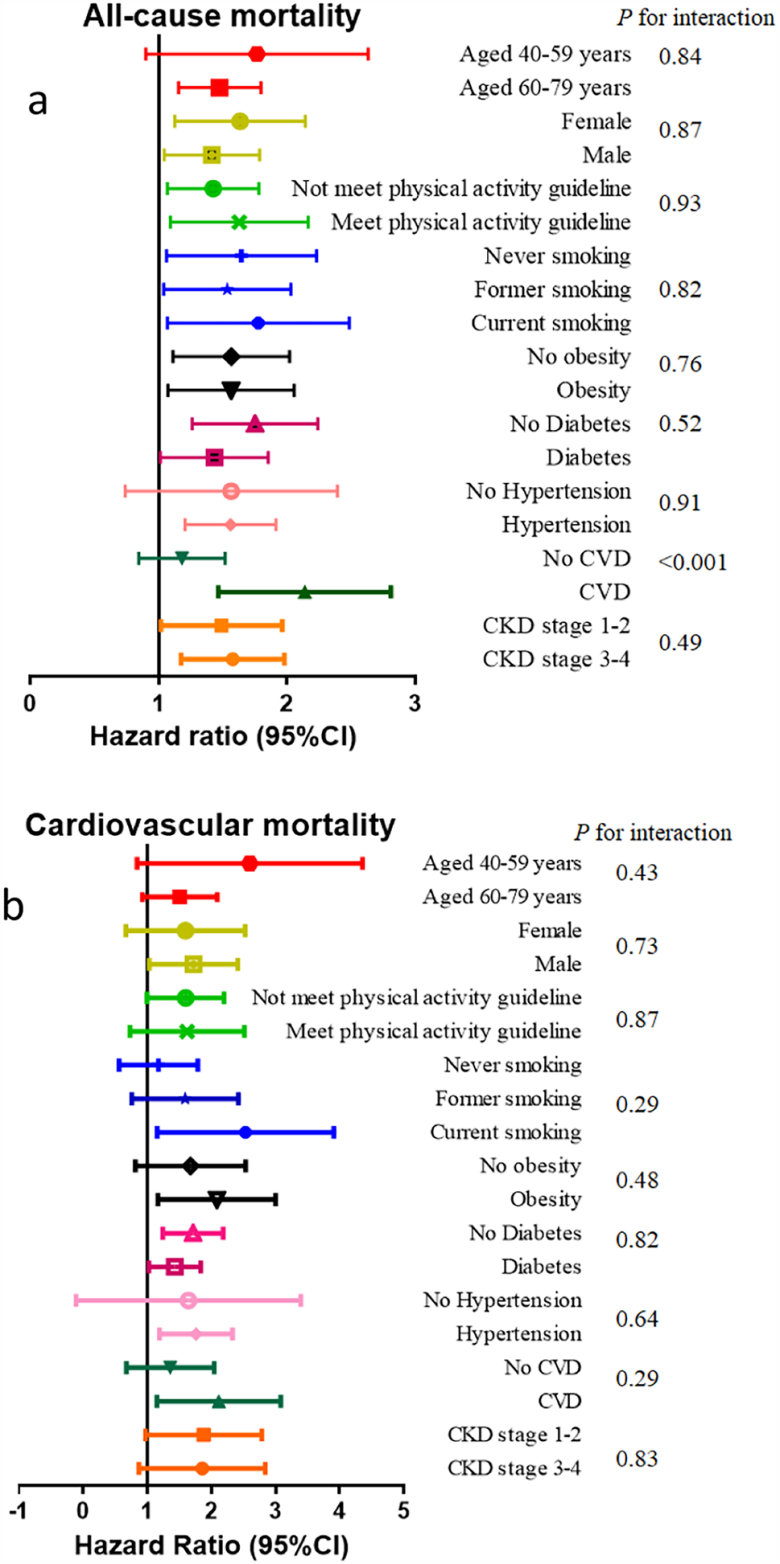
Subgroup analysis: Association between serum Klotho levels and all-cause mortality and cardiovascular mortality. *Note*: The cut-off values of Klotho are 548.8 pg/ml for all-cause mortality (**a**) and 660.9 pg/ml for cardiovascular mortality (**b**), respectively.

We did not observe the interactions between Klotho and age, sex, physical activity, smoking, obesity, diabetes, hypertension and CKD stages for all-cause and cardiovascular mortality (Fig. 6). However, a significant interaction between Klotho and CVD were identified for all-cause mortality (P for interaction < 0.001). Patients with CVD had a higher risk of all-cause mortality with a cut-off value of Klotho < 548.8 pg/ml compared to those without CVD (HR and 95% CI 2.07 (1.50, 2.84) vs. 1.15 (0.86, 1.53)). The association between serum Klotho concentration and all-cause and cardiovascular mortality was consistent in subgroups according to age (40–59 vs. 60–79 years old), sex, physical activity (meet the suggested guideline or not), smoking (never, former or current), obesity, diabetes, hypertension, CVD and CKD stages (stage 1–2 vs. stage 3–4).

## Discussion

Based on a national-level CKD subgroup of the NHANES survey, we identified the cut-off values of Klotho and evaluated its effects on all-cause and cardiovascular mortality in pre-dialysis CKD patients. Klotho < 548.8 pg/ml was associated with 52% higher risk of all-cause mortality, and Klotho < 660.9 pg/ml was associated with 58% higher risk of cardiovascular mortality after adjusting for multiple confounders.

The kidney is the main source of circulating Klotho protein, which is highly expressed in distal convoluted tubules. Klotho is severely downregulated in CKD^23^. The median concentration of Klotho in general population of NHANES is 810 pg/ml^9^. Whereas, this study showed that the median concentration of Klotho is 760 pg/ml in CKD patients who have not entered ESKD, and it reduced as renal function deteriorates. Population studies also indicate that low levels of Klotho act as a predictor of CKD progression rather than just a biomarker of kidney injury^15,24^. The causes of Klotho loss in CKD including loss of viable tissue, abnormal mineral metabolism, hypermethylation, or deacetylation of the Klotho gene promoter, as well as oxidative stress, inflammation, the renin-angiotensin system, and the effect of uremic toxins^3^. However, the details of these potential mechanisms remain largely unclear.

Although previous studies reported that Klotho is related to atherosclerosis and longevity in mice^2,25^, there is a paucity of studies on the association between Klotho levels and cardiovascular mortality in human populations, and the results are conflicting^9,10,12^. To the best of our knowledge, there are no previously studies explored the cut-off values of Klotho for all-cause and cardiovascular mortality in pre-dialysis CKD. Only one study reported a threshold of 574 pg/ml and 465 pg/ml for predicting all-cause and cardiovascular mortality by using a two-piecewise linear regression model, and indicated that lower serum Klotho concentrations were associated with higher risk of all-cause mortality, but not cardiovascular mortality in hypertensive patients^10^. However, our results found that Klotho was independently associated with cardiovascular mortality in patients with CKD and was more pronounced in patients with hypertension. In addition, increasing evidence has shown that a lower Klotho concentration is an independent predictor of all-cause and cardiovascular mortality and can accelerate coronary artery calcification in patients on maintenance hemodialysis^13,26,27^. Unfortunately, patients were classified based on mean/median/tertile/quartile of Klotho levels with various reference range in these regional and small sample studies without reporting a threshold^11,12,24,26–28^. In this study, we observed a non-linear relationship between Klotho and all-cause and cardiovascular mortality in CKD patients. Notably, the risk of all-cause and cardiovascular mortality gradually increased as Klotho concentration decreased when below 700 pg/ml. Furthermore, ROC curves revealed the optimal cut-off values of Klotho are 548.8 pg/ml and 660.9 pg/ml for all-cause and cardiovascular mortality, respectively. Experimental results have indicated that the Klotho protects the heart from myocardial hypertrophy by inhibiting transient receptor potential channel-6 activity in cardiomyocytes^29^. Myocardial hypertrophy was alleviated after injecting a transgene encoding Klotho intravenously in Klotho-deficient CKD mice^30^. Our study provided new evidence on the threshold of Klotho for intervention in the future.

This study’s strengths include the relatively large sample size with CKD based on a national sample of the American population, as well as a rigorous cause-of-death data record. Furthermore, our study had comprehensive information on confounders such as demographics, living habits, comorbidities, and medications, as well as laboratory data such as mineral metabolism. This study also had several limitations that should be mentioned. Firstly, the definition of CKD we used is based on a single measurement of the eGFR and ACR despite the guideline for diagnosis recommending repeated testing. However, the definition of CKD is also based on a time point measurement of the eGFR in previously published studies^31^. Secondly, the study population was limited to participants aged 40–79 years, and may limit the generalizability of the findings to patients of other-age-ranges. However, patients with CKD are usually older population. Thirdly, we were unable to include FGF23, which plays a role in the FGF23/Klotho axis and is related to mineral metabolism as a confounder. However, we adjusted the FGF23/Klotho axis-related minerals, including calcium, phosphorus, and 25(OH)D, in the Cox regression model. Finally, the possibility of residual confounders could not have been ruled out.

In summary, cardiovascular or any cause death should be earlier-forecast when Klotho < 700 pg/ml in CKD patients. The risk of cardiovascular mortality increased especially when Klotho < 660.9 pg/ml, and the risk of all-cause mortality was further increased when Klotho < 568.8 pg/ml. Monitoring Klotho concentrations when below 700 pg/ml are necessary for preventing premature death in patients with CKD. Further studies are warranted to verify our findings. It is also urgent to seek targets that increase Klotho concentrations and strive to achieve clinical transformation.

## Data Availability

The datasets analyzed during the current study are available in the National Health and Nutrition Examination Survey (NHANES) website: https://www.cdc.gov/nchs/nhanes/index.htm.
